# Evaluation of Anti-inflammatory and Analgesic Activity of the Extract and Fractions of *Astragalus hamosus* in Animal Models 

**Published:** 2015

**Authors:** Asie Shojaii, Majid Motaghinejad, Sima Norouzi, Manijeh Motevalian

**Affiliations:** a *Department of Pharmacology, School of Medicine and Razi Drug Research Center, Iran University of Medical Sciences, Tehran, Iran. *; b *Research Institute for Islamic and Complementary Medicine, Iran University of Medical Sciences, Tehran, Iran.*

**Keywords:** Traditional Iranian Medicine, Analgesic, Anti-inflammatory, *Astragalus hamosus*

## Abstract

The objective of this study was to evaluate the anti-inflammatory and analgesic activities of the hydro-alcoholic extract of the pods of *Astragalus hamosus *(HAAH), a plant used in Iranian traditional medicine, and antinociceptive effects of different fractions in animal models. The anti-inflammatory effect was evaluated by the rat paw edema induced by formalin. Also the analgesic effect was examined by the acetic-acid-induced writhing response and hot plate test. The analgesic effects of chloroform, hexane, ethyl acetate and aqueous fractions were evaluated by the hot-plate method. The hydroalcoholic extract of *Astragalus hamosus* could reduce the edema in a dose-dependent manner (P<0.05). In the acute phase, the result of 1000 mg/Kg and in the chronic phase, the result of 100 and 300 mg/Kg of the extract were more significant and comparable with the effect of sodium salicylate. Also application of different doses of HAAH had significant anti-nociceptive effects on both animal models. The findings showed that HAAH at doses of 700 and 1000 mg/Kg produced analgesic effects comparable to sodium salicylate. The hexane and ethyl acetate (but not the other fractions) showed significant analgesic activity in hot plate test, when compared to morphine. The results of this study demonstrated the anti-inflammatory and analgesic effects of HAAH extract and hexane and ethyl acetate fractions of the extract in animal models and justify traditional use of this plant in the treatment of pain and inflammatory conditions. More studies to clarify the active components are necessary.

## Introduction

Inflammation and pain are common nonspecific manifestations of many diseases. Although non-steroidal anti-inflammatory drugs (NSAIDs) and opiates have been used classically in these conditions, but some adverse reactions occur with these drugs such as gastrointestinal disturbances, renal damage, respiratory depression, and possible dependence (1-2). In recent years, there has been an increasing interest to find new anti-inflammatory and analgesic drugs with possibly fewer side effects from natural sources and medicinal plants.

*Astragalus hamosus* (known as nakhonak in Iran) is a plant belonging to family leguminosae, which has been used traditionally for treatment of painful and inflammatory conditions. Also it used for treatment of some nervous diseases in Iranian traditional medicine (3). Genus Astragalus comprising nearly 3000 species all around the world, and 800 annual and perennial species of it were found in Iran (4-5). *Astragalus hamosus* is an annual plant with cylindrical and arch-shape yellow to brownish fruits which grows in arid and desert areas of Iran like Kashan, Khozestan and Boushehr (6). Pharmacological evaluations have shown antioxidant activity of methanolic extract of *Astragalus hamosus* (7). Also, volatile compounds of this plant showed significant cytotoxic activity against human acute lymphoid leukemia in concentration-dependent manner (8). Evaluation of ant proliferative effect of a flavonol glycoside and saponins of *Astragalus* *hamosus* by MTT-dye reduction assay showed concentration-dependent inhibition of malignant cell proliferation by saponins, while the flavonoid exerted only marginal effects (9). In a preliminary study on 2010, only acute Anti-inflammatory activity of the methanol extract of *Astragalus hamosus* has been reported (10).

Based on Iranian folk medicine, in the present study acute and chronic anti-inflammatory and analgesic effects were evaluated for HAAH and analgesic effect of its fractions in rat using formalin induced inflammation, acetic-acid writhing test and hot plate respectively.

## Experimental

*Plant material*

The fruits of *Astragalus hamosus* were purchased from a local medicinal plant shop in Tehran and identified by M.Kamalinejed (School of Pharmacy, Shahid Beheshti University of medical sciences, Tehran, Iran), the voucher specimen (8005) was deposited in School of Pharmacy. 

*Preparation of extract and fractions*

For preparation of the hydro-alcoholic extract, 300 g of dried and grinded pods of *Astragalus hamosus *were macerated in ethanol 70% for three times (each time 24 h). The extract was then filtered and concentrated with vacuum evaporator and the percentage yield was 13%.

To yield different fractions, dried hydro alcoholic extract was suspended in water and partitioned by hexane, chloroform and ethyl acetate (each solvent in duplicate). Each fraction was evaporated to obtain hexane fraction (42 g), chloroform fraction (10 g), ethyl acetate fraction (4 g) and water fraction (25 g) which were used for bioassay.

*Phytochemical screening*

Phytochemical investigations of the HAAH were carried out using standard methods and tests (11-13). The test for tannins was carried out by subjecting 1 g of extract in 2 mL of distilled water, filtered and ferric chloride reagents were added to the filtrate. The extract was subjected to frothing test for the identification of saponins and to Fehling's test for glycosides. Alkaloids were detected in the alkaloid fraction obtained by a classical acid: base extraction procedure for alkaloids and analyzed by TLC in chloroform: methanol: ammonia solution 25% 8:2:0.5 as solvent system, spots were detected after spraying with Dragendorff’s reagent. The presence of flavonoids was determined using 1% aluminum chloride solution to the extract and yellow coloration. Another test for flavonoid, dilute ammonia (5 mL) was added to the extract and then concentrated sulphuric acid (1 mL) was added. Steroids were detected by adding 1 mL of acetic anhydride to 0.25 g methanolic extract of each sample with 1 mL H_2_SO_4_. The color changed from violet to blue or green indicating the presence of steroids. The test for anthraquinones was performed with 0.5 g of extract boiled with 10 mL sulphuric acid and filtered. Then filtrate was shaken with 5 mL CHCl_3_ and CHCl_3_ layer was removed to another tube and 1 mL of ammonia was added and colour change was observed. Detection of terpenoids (triterpenoids) was carried out by adding 2 mL of CHCl_3_ to 0.5 g of extract and then adding carefully concentrated H_2_SO_4_ (3 mL) to form a layer and reddish to brown color in interface.

*Animals*

42 adult male wistar rats (150-200 g) and 77 adult male albino mice (25-35 g) were housed in animal unit of Iran University of Medical Sciences under standard laboratory conditions (temperature 23 ± 2 ˚C) with 12 h dark and 12 h light cycle. The animals had free access to standard dry pellet diet and tap water ad libitum.

*Anti-inflammatory activity*

*Formalin induced rat paw edema*

The test was carried out using the method described by Hunskaar and Hole (14)**.**

Initially, 42 adult wistar rats were divided into 6 groups. The animals in each group were treated with HAAH at doses of 100, 300, 700 and 1000 mg/Kg *i.p*. and 300 mg/Kg sodium salicylate as positive control group and 1 mL distilled water as negative control group according to our previous studies. Then the rat paw edema induced by injection of 50 µL of 2.5% formalin (in normal saline 0.9%) into sub-planar tissue of the paws of rats and paw volumes were measured by plethysmometer during 8 days after formalin injection. Animals were treated with plant extract and drugs every day. Then the percentage inhibition of edema was calculated by the following formula:

Percentage Inhibition= ((Vt-V0)/V0) ×100

Vt= volume of animals’ paw after injection

V0=volume of animals’ paw before injection

*Analgesic activity*

*Acetic acid-induced writhing test*

The acetic-acid writhing test was performed using the reported procedure (15).

Groups of rats (n=7), were administered with100, 300, 700, 1000 mg/Kg of HAAH *i.p*., 300 mg/Kg sodium salicylate as positive control group and 1 mL distilled water as negative control group. After 30 minutes the animals were administered with i.p. injection of 0.1 mL acetic acid (0.6%). Then the count of abdominal contractions of animals during 30 minutes after acetic acid injection was reported and the Percentage Analgesic Activity (PAA) was calculated by using the following formula:

PAA = ((C- C_D_)/C_D_) ×100

C = Mean of contractions’ count in animals treated with different doses of *Astragalus* *hamosus* extract and sodium salicylate 

C_D _= Mean of contractions’ count in animals served as negative control

*Hot plate test*

The Hot plate test was performed using the reported procedure (1).

Anti-nociceptive effect of HAAH and its fractions was investigated using hot plate test in seventy-seven adult male albino mice. The same procedure was applied to the animals of each group and the latency time was measured and compared to control group. The Percentage Analgesic Activity (PAA) was calculated by using the following formula:

PAA= ((L_a_-L_b_)/L_b_) ×100

L_a _=Latency time after treatment with drug or extract 

L_b_=Latency time before treatment with drug or extract

The analgesic effects of different fractions of HAAH were also evaluated with the same procedure. Morphine used as positive control. 

*Statistical analysis*

The results are reported as mean ± S.E.M. The statistical analyses were performed using one way analysis of variance (ANOVA). Group differences were calculated by post hoc analysis using Tukey’s test. For all tests, differences with values of P<0.05 were considered significant.

## Results

Preliminary phytochemical study of the hydro alcoholic extract of *Astragalus* *hamosus*, showed the presence of saponins, terpens, phenols, alkaloids, tannins and flavonoids.

*Formalin-induced inflammation*

This study showed that the HAAH could reduce the rat paw edema in a dose-dependent manner (P<0.05). At second hour after the extract injection, the percentage inhibition of edema was found to be 60.88 % to 76.01% for different doses of extract (Table 1). In acute phase of inflammation, the results revealed a significant anti-inflammatory activity for HAAH at 1000 mg/Kg dose which was comparable to sodium salicylate.

**Table 1:** Evaluation of anti-inflammatory effects of *Astragalus hamosus* extract (Acute inflammation).

**p-value**	**Percentage Inhibition (%)**	**Increase in volume of rat paw ( mm** ^3^ **) **	**Volume of rat paw (mm** ^3^ **)2 h after injection**	**Volume of rat paw ( mm** ^3^ ** )Before injection**	**Dose(mg/kg)**	**Groups**
.94	60.88%	.440±.05	1.58±.07	1.14±.05	100	HAAH
.58	62.42%	.397±.02	1.46±.04	1.06±.03	300	HAAH
.99	58.66%	.468±.04	1.60±.05	1.13±01	700	HAAH
.002	76.01%	.241±.01	1.24±.03	1.00±.01	1000	HAAH
.01	73.09%	.282±.01	1.34±.02	1.05±.02	300	Sodium salicylate
_	56.38%	.500±.04	1.64±.06	1.14±.02		Distilled water

Values are presented as Mean±SEM (n=7 in each group)

The results of the formalin test showed that the hydro alcoholic extract of *Astragalus hamosus *possesses significant anti-inflammatory effects in the chronic phase of inflammation. The results of extract in doses 100 and 300 mg/Kg were more significant and comparable with sodium salicylate (Table 2).

**Table 2:** Anti-inflammatory effects of hydro alcoholic extract of *Astragalus hamosus* (HAAH) on formalin induced edema in rats (Chronic inflammation).

**Changes in mean paw edema (mL)**										
**9 days**	**8 days**	**7 days**	**6 day** **s**	**5 days**	**4 days**	**3 day** **s**	**2 days**	**1 day**	**Dose(mg/Kg)**	**Groups **
±.05 0.09	±.05 0.11	±.06 0.17	±.05 0.17	±.04 0.21	.06 ± 0.28	±.06 0.35	±.09 0.53	0.25±.02	100	HAAH
±.04 0.15	±.04 0.21	±.04 0.25	±.04 0.27	±.05 0.27	±.05 0.28	±.05 0.32	±.04 0.39	0.24 ±.04	300	HAAH
±.02 0.13	±.03 0.16	±.04 0.22	±.03 0.28	±.04 0.36	±.05 0.43	±.05 0.53	±.05 0.68	±.03 0.31	700	HAAH
±.03 0.34	±.03 0.37	±.02 0.39	±.02 0.40	±.02 0.42	±.02 0.47	±.02 0.53	±.02 0.58	±.02 0.17	1000	HAAH
±.03 0.10	±.02 0.16	±.01 0.20	±.00 0.24	±.01 0.27	±.04 0.33	±.06 0.45	±.07 0.53	±.01 0.2	300	Sodium salicylate
±.02 0.31	±.03 0.36	±.03 0.41	±.02 0.42	±.03 0.49	±.04 0.57	±.04 0.67	±.06 0.72	±.03 0.36		Distilled water

Values are presented as mean±SEM (n=7 in each group), P<0.05

*Acetic acid-induced writhing response*

**Figure 1:**
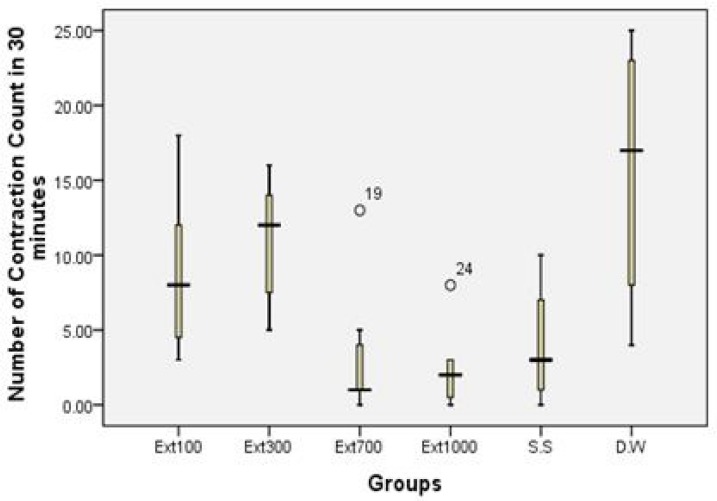
The boxplot showing the writhing response in 30 minutes after acetic-acid injection in the presence of different doses of HAAH in experimental animals. S.S = Sodium Salicylate, D.W = distilled water (n=7 in each group).

The second study showed that the application of different doses of HAAH had significant analgesic effects in the animals under investigation. The results of doses 700 and 1000 mg/Kg were significant and comparable with the effect of sodium salicylate in analgesic activity (Table 3 and Figure 1).

**Table 3:** Effects of ethanol extract of *Astragalus hamosus* on acetic acid–induced writhing response (N=7 in each group).

p-value	Percentage	(number of writhing movements) (Mean ± S.E)	Groups
.36	43.48%	8.85 ± 2.15	Extract 100
.72	30.71%	10.85 ± 1.62	Extract 300
.009	78.16%	3.42 ± 1.71	Extract 700
.004	84.54%	2.42 ± 1.04	Extract 1000
.02	74.45%	4:00 ± 1.57	Sodium Salicylate
_	_	15.66 ± 3.40	Distilled water

*Hot plate test*

The findings of hot plate test showed that the HAAH possesses significant anti-nociceptive effect in comparison to control group. The result of 1000 mg/Kg was significant and comparable to sodium salicylate (Table 4)**.**

Evaluation of the analgesic activity of different fractions of HAAH showed that Ethyl acetate and hexane fractions had significant analgesic effects compared to morphine (p<0.05) (Table 4).

**Table 4:** Evaluation of anti-nociceptive effects of *Astragalus* *hamosus*hydro- alcoholic extracte and its fractions in hot plate test (N=7 in each group).

**Percentage analgesic activity(PAA) in hot plate test ** **Time after injection (min)**				**Dose(mg/Kg)**	**Groups**
30 60 120 180					
15.66 ± 0.90	15.70 ± 13.58	23.82 ± 1.69	20.00 ± 8.86	100	HAAH
22.11 ± 39.55	16.85 ± 21.96	17.62 ± 40.24	28.536 ± 6.26	300	HAAH
14.91 ± .56	20.88 ± 8.74	16.99 ± 21.30	13.78 ± 2.96	700	HAAH
38.87 ± 89.59	13.733 ± 8.77	22.899 ± 9.48	30.539 ± 2.41	1000	HAAH
19.5 ± 4.7	38.4 ± 4.3	51.7 ± 11.2	15.7 ± 9.0	10	Morphine
35.4 ± 23.13	44.3 ± 25.6	45.15 ± 22.3	18.05 ± 14.6	1000	Hexane fraction
5.3 ± 9.5	1.4 ± 14.2	4.70 ± 3.3	6.1 ± 1.9	1000	CHCl_3_ fraction
22 ± 18.3	26.2 ± 16.8	46.0 ± 25.7	13.2 ± 11.8	1000	EtOAc fraction
39.9 ± 40.5	10 ± 12.9	8.7 ± 8	5.7 ± 8.8	1000	Aqueous fraction
52.60 ± 72.24	64.481 ± 16.41	90.961 ± 46.82	22.045 ± 3.97	300	Sodium Salicylate
9.15 ± 28.19	8.27 ± 36.49	6.49 ± 33.80	9.11 ± 30.72		Distilled Water

HAAH=hydroalcholic extract of *Astragalus hamosus*.

Data presented as Mean±SEM

*Discussion and conclusion*

In traditional medicine of Iran, several natural products have been used to treat pain and inflammation. *Astragalus hamosus* is a plant used for treatment of painful and inflammatory conditions in Iranian traditional medicine for many years (3). In this study, anti-inflammatory and analgesic activities of the hydro-alcoholic extract (70%) of the pods of *Astragalus hamosus* were assessed in different well accepted animal models, including formalin-induced rat paw edema, acetic acid-induced writhing test and hot plate test. In order to further evaluate the effective components of the plant, the antinociceptive effect of different fractions of HAAH were assessed by hot plate test.

Formalin-induced rat paw edema is a well known and standard experiment phase which develops in a few hours is attributed to the release of histamine, serotonin and kinins (16-18). The chronic phase is accompanied by the release of prostaglandins (19). Since activity was observed in both phases of formalin-induced edema, possible activity might be due to the inhibition of release of several mediators such as histamine, serotonin, kinins and prostaglandins (Tables 1 and 2). Although the effect on chronic phase is more pronounced and this is probably indicative of the inhibitory effects of HAAH on prostaglandin synthesis or release, these results confirmed the preliminary work reported previously (10).

The analgesic activity was assessed by writhing test which has been reported to be useful for investigation of peripheral antinociceptive activity and performed as a chemical pain model (20-21). The hot plate test was performed as a thermal pain model which is known useful for study of the central mechanism of analgesic activity.

The ethanol extract of *Astragalus hamosus *demonstrated a dose-dependent, significant antinociceptive activity in both animal models of pain. Acetic acid believed to increase the PGE_2_ and PGF_2α_ in peritoneal fluid (9). However, in hot plate model, the extract in different doses increased the pain threshold centrally. Therefore, the analgesic activity shown in two models of pain is indicative that HAAH might possess centrally and peripherally mediated antinociceptive properties.

According to the results of the hot plate test, the hexane and ethyl acetate fractions showed a significant analgesic activity in animal model. Nevertheless, the chloroform and aqueous fractions of HAAH have shown no significant analgesic effect in hot plate test (Table 4).

Phytochemical screening of HAAH revealed the presence of considerable quantities of flavonoids, saponins, terpens, alkaloids, tannins and phenols. Several reports have shown the analgesic and anti-inflammatory properties of flavonoids, tritrepenoids, tannins and other polyphenolic compounds in different experimental animal models (9, 22-25). Moreover, tritrepenoids, flavonoids and tannins are known to inhibit prostaglandin synthesis and the effect of HAAH in chronic phase of inflammation could be attributed to inhibition of prostaglandin release due to the presence of these components.

So far different chemical compounds including flavonoids and saponins were isolated from *Astragalus hamosus*. Some of these compounds showed biological effects such as antiproliferative and modulators of lymphocyte proliferation (8, 23). A new flavonol glycoside 7-O-methyl-kaempferol 4'-beta-D-galactopyranoside (rhamnocitrin 4'-beta-D-galactopyranoside) was isolated from the aerial parts of *Astragalus hamosus*. The known flavonols hyperoside, isoquercitrin and astragalin were also identified. Structures of the compounds were elucidated by chemical and spectral methods (22).

Chemical components of HAAH extract such as flavonoids, saponins or phenolic compounds may be responsible for the antinociceptive and anti-inflammatory activities of this plant. Since the findings of this study revealed a significant analgesic effect of the hexane and ethyl acetate fractions of HAAH extract, it can be concluded that terpenoids and specially saponins of *Astragalus hamosus* may be responsible for the observed analgesic effect which should be proved by further investigations. 

It can be concluded that hydroalcoholic extract of the *Astragalus hamosus* possesses anti-nociceptive and anti-inflammatory properties which are probably mediated via inhibition of prostaglandin synthesis as well as central inhibitory mechanisms which may be of potential benefit for the management of pain and inflammatory disorders.
